# Moyamoya Disease in Pregnancy: A Case Series

**DOI:** 10.1055/a-2930-3966

**Published:** 2026-08-20

**Authors:** Hannah Elizabeth McCool, Nick Cochran, Kerri Spontarelli Fruit, Bill Atkinson

**Affiliations:** 1 Department of Obstetrics and Gynecology 12343 Texas Tech University Health Sciences CenterLubbockTexasUnited States; 2 12343 Texas Tech University Health Sciences Center School of MedicineLubbockTexasUnited States

**Keywords:** moyamoya disease, pregnancy, preeclampsia, intracerebral hemorrhage, cerebral revascularization, hypertensive disorders of pregnancy, delivery planning

## Abstract

**Objective** Moyamoya disease is a rare progressive cerebrovascular disorder characterized by stenosis of the intracranial internal carotid arteries and formation of fragile collateral vessels, predisposing patients to ischemic and hemorrhagic stroke. Physiologic changes of pregnancy—including increased blood volume, systemic vasodilation, and augmented cerebral blood flow—may increase cerebrovascular vulnerability and complicate management.

**Study Design** We describe three pregnancies complicated by moyamoya disease managed at a tertiary referral center in West Texas. All patients had undergone prior cerebral revascularization and were followed by neurology before conception. Antithrombotic strategies varied by individual risk factors and included aspirin or low-molecular-weight heparin. Comorbidities included pregestational diabetes with dichorionic diamniotic twins, homozygous factor V Leiden, and Chiari malformation with MTHFR mutation.

**Results** Two patients underwent planned cesarean delivery with favorable maternal and neonatal outcomes following multidisciplinary management. The third developed preeclampsia with severe features at 32 weeks and experienced sudden neurologic deterioration. Despite emergent cesarean delivery, she suffered a fatal intracerebral hemorrhage.

**Conclusion** These cases highlight the spectrum of pregnancy outcomes in patients with moyamoya disease and emphasize the importance of multidisciplinary care, strict blood pressure control, and careful neurologic surveillance.

**Key Points**

- Rigorous blood pressure control is essential in pregnant patients with moyamoya disease due to increased risk of intracerebral hemorrhage during hypertensive crises.
- Multidisciplinary care—including neurology, maternal–fetal medicine, and anesthesia—optimizes maternal and neonatal outcomes.
- Delivery planning should minimize hemodynamic stressors; however, operative vaginal and cesarean deliveries appear to have similar stroke outcomes, and mode should be individualized.

## Introduction

Moyamoya disease is a progressive cerebrovascular arteriopathy characterized by stenosis of the intracranial internal carotid arteries and the development of fragile collateral vessels. Although surgical revascularization reduces recurrent ischemic events, patients remain at risk for both ischemic and hemorrhagic complications. Pregnancy introduces additional physiologic stressors—including increased circulating blood volume, reduced systemic vascular resistance, and heightened cardiac output—that may alter cerebral perfusion dynamics. Furthermore, hypertensive disorders of pregnancy can further destabilize fragile collateral vasculature, increasing the risk of intracranial hemorrhage. 1

Given the rarity of moyamoya disease in pregnancy, standardized obstetric management strategies are lacking. Decisions regarding anticoagulation, blood pressure control, and delivery planning are often individualized and guided by limited data. 2 We present three pregnancies complicated by moyamoya disease following prior revascularization, illustrating the range of maternal outcomes and highlighting key management considerations.

## Case 1

Patients with moyamoya disease who have a history of multiple prior strokes and significant residual deficits require particularly careful pregnancy management, especially when additional metabolic or obstetric comorbidities are present.

A 37-year-old G3P1011 presented at 6 weeks’ gestation with a history of moyamoya disease diagnosed after four prior strokes involving the left occipital, parietal, and temporal lobes, corpus callosum, and bilateral frontal subcortical regions. Residual deficits included mild left leg weakness, right homonymous hemianopia, and speech and memory impairment. She underwent bilateral superficial temporal artery–middle cerebral artery (STA-MCA) bypasses in 2021, with her last stroke in February 2023. Comorbidities included class B pregestational diabetes, migraine headaches, and prior cesarean section.

**Fig. 1:**
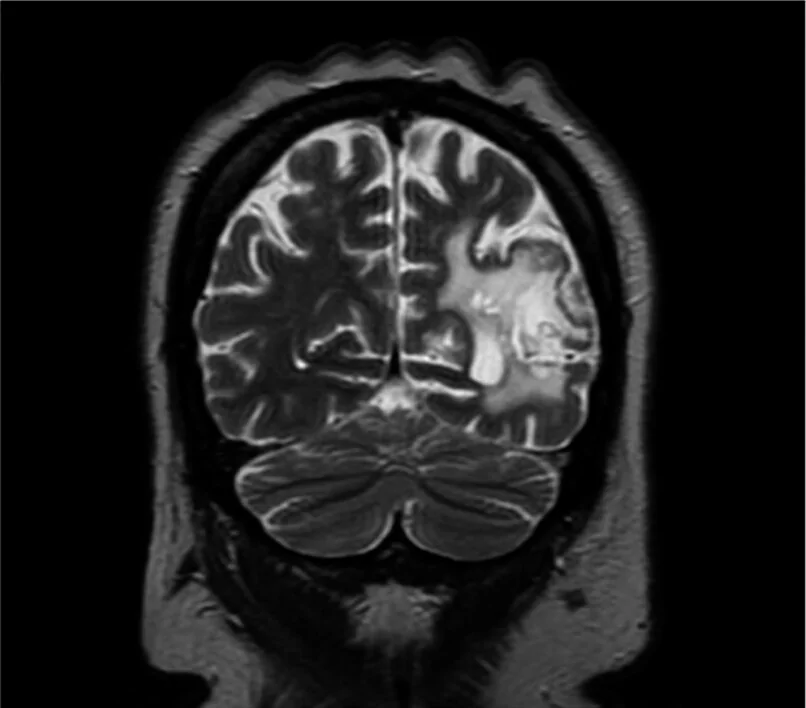
Magnetic resonance imaging (MRI) of the brain in Case 1 demonstrating chronic multifocal ischemic changes involving the left occipital, parietal, and temporal lobes, as well as bilateral frontal subcortical regions, without evidence of acute infarction.

At 11 weeks, ultrasound confirmed a dichorionic diamniotic twin pregnancy. She continued aspirin 81 mg daily and insulin therapy, which was adjusted frequently throughout pregnancy for glycemic control. Her course was complicated by recurrent hospital admissions for hyperglycemia and intractable headaches. Magnetic resonance imaging (MRI)/magnetic resonance angiography (MRA) at 17 weeks showed chronic ischemic changes without acute infarction ( Fig. 1 ). At 30 weeks, she was readmitted for disorientation, with imaging again showing no new lesions. At 34 weeks, she developed preeclampsia without severe features, characterized by elevated blood pressures and a 24-hour urine protein of 1,120 mg. At 34 ^5/7^ weeks, due to severe abdominal pain and a category II fetal heart tracing, she underwent a repeat low transverse cesarean section. Both infants were delivered safely and admitted to the NICU for prematurity and respiratory support. Her postoperative course was uncomplicated.

## Case 2

When moyamoya disease coexists with inherited thrombophilias such as factor V Leiden, managing pregnancy care becomes more complex due to overlapping risks of arterial ischemia, venous thrombosis, and hypertensive complications.

A 34-year-old G3P0111 presented for prenatal care at 10 ^2/7^ weeks of gestation. Her medical history was significant for moyamoya disease, homozygosity for factor V Leiden, and severe preeclampsia in her first pregnancy. She also experienced a spontaneous twin pregnancy loss in her second gestation. Moyamoya disease was diagnosed in childhood following a right middle cerebral artery stroke at the age of 4, with subsequent right STA–MCA bypass in 1995 and encephalomyosynangiosis in 2007. She had no history of hemorrhagic stroke or venous thrombosis.

**Fig. 2:**
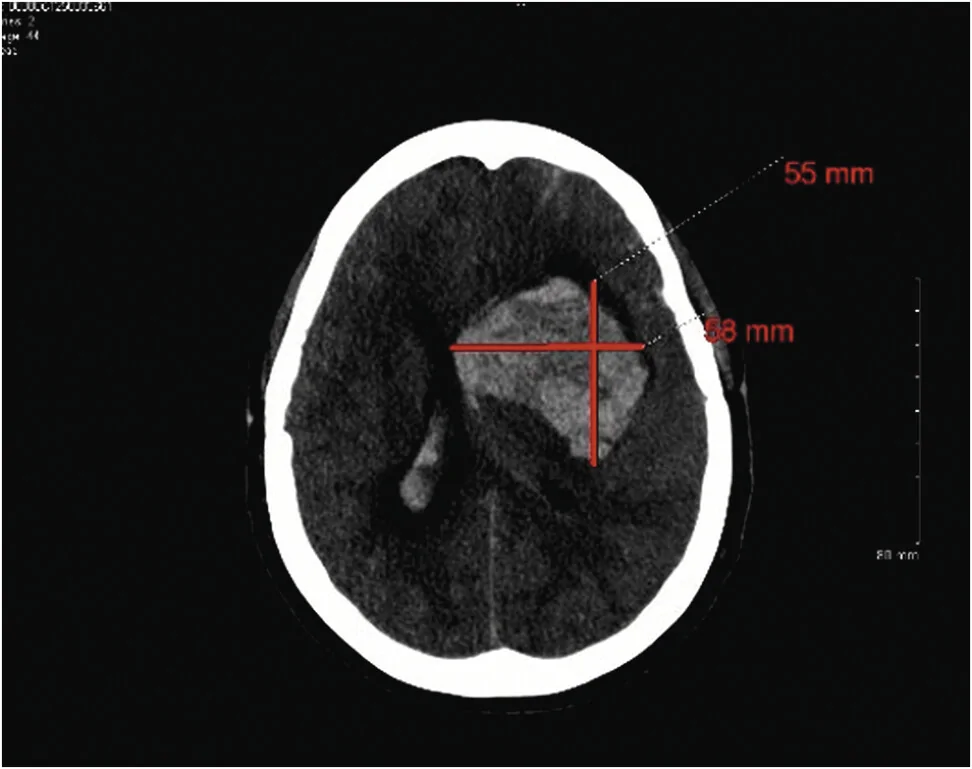
Computed tomography (CT) of the head in Case 2 demonstrating a large left intraparenchymal hemorrhage with surrounding edema, midline shift, and evidence of transtentorial herniation.

**Fig. 3:**
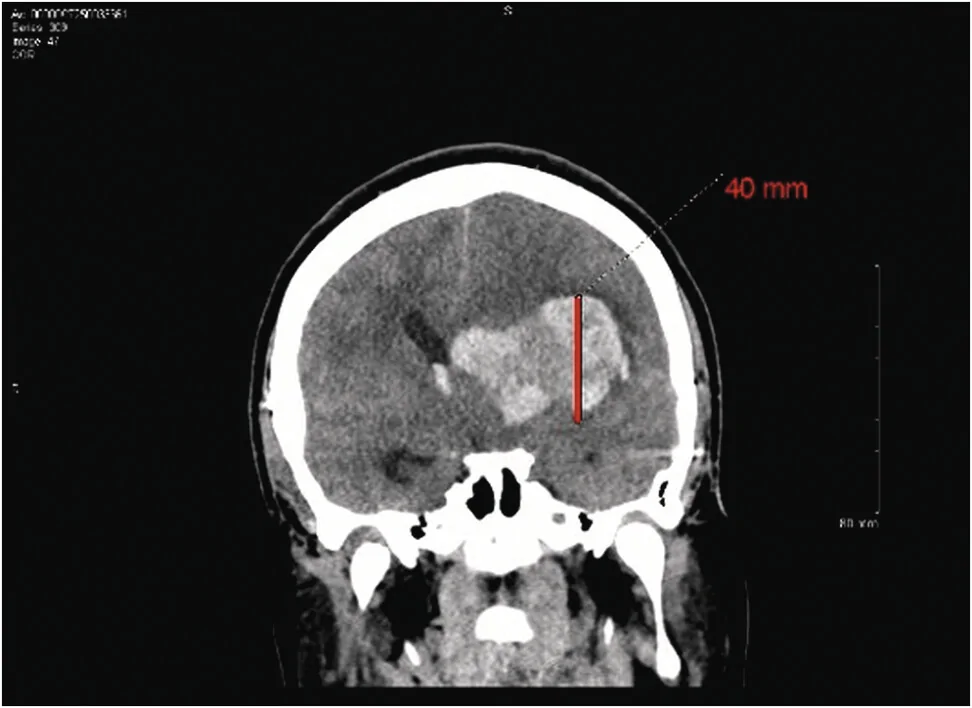
Computed tomography (CT) angiography of the brain in Case 2 demonstrating active arterial extravasation within the left cerebral hemisphere, consistent with ongoing hemorrhage.

Daily medications included aspirin 325 mg and prenatal vitamins. Her most recent preconception MRA (2024) demonstrated remote right-sided watershed infarcts, which were stable compared with prior imaging as per her neurology team’s assessment. Following preconception counseling with maternal–fetal medicine (MFM), she conceived through frozen embryo transfer and continued aspirin monotherapy for thromboprophylaxis. The patient developed gestational hypertension at 22 weeks, which was initially managed with nifedipine; however, this was changed to labetalol due to the patient’s inability to tolerate side effects of nifedipine. She was diagnosed with superimposed preeclampsia at 31 weeks due to an elevated urine protein–creatinine ratio, and was admitted to the antepartum service at 32 weeks for increased swelling and elevated blood pressures reported on a home log. During this admission, the patient received antenatal corticosteroids with delivery planning at 34 weeks via cesarean section. Her blood pressure remained stable on her home regimen; however, on hospital day 5, she developed a sudden, severe headache in the absence of other symptoms. Magnesium sulfate was initiated for seizure prophylaxis, given her new diagnosis of preeclampsia with severe features. Initially, the patient had improvement in her symptoms, but after discussion with providers and the patient, the plan was changed to proceed toward cesarean delivery later that day. Prior to the scheduled surgery, the patient experienced worsening symptoms of her headache and subsequently was found to be unresponsive with decerebrate posturing. An emergent cesarean section was performed, delivering a 1,900 g male infant with Apgar scores of 8 and 9. Postoperatively, computed tomography (CT) imaging revealed a large left intraparenchymal hemorrhage with active arterial extravasation, left frontal infarction, midline shift, and herniation through the foramen magnum ( Figs. 2 and 3 ). Neurosurgical consultation concluded the injury was non-survivable. After family discussions, care was transitioned to comfort measures, and the patient passed away on postoperative day 2.

## Case 3

Pregnancy in patients with moyamoya disease poses additional risks from physiologic hypervolemia, altered vascular tone, and hypertensive disorders. While they often remain neurologically stable, delivery planning must still account for hemodynamic stressors and individualized stroke-prevention strategies.

A 22-year-old G2P1001 presented at 16 ^3/7^ weeks of gestation with a history of moyamoya disease, Chiari malformation, and MTHFR mutation with normal homocysteine levels. She was diagnosed with moyamoya disease at the age of 15 after presenting with transient ischemic symptoms, and underwent bilateral STA–MCA bypass at the age of 16 with near-complete neurologic recovery. She has remained stable since 2017 on aspirin 81 mg daily.

Her first pregnancy resulted in a forceps-assisted vaginal delivery at 38 weeks to minimize maternal Valsalva effort. To help reduce her risk of thromboembolism, she was started on prophylactic Lovenox 40 mg daily, consistent with management from her prior pregnancy. She remained asymptomatic throughout the pregnancy without neurologic changes or headaches. At 35 weeks, induction and delivery planning were discussed, with the goal of minimizing maternal effort during the second stage. At 37 weeks, the fetus was found to be breech, and the multidisciplinary team recommended a primary low transverse cesarean section rather than external cephalic version. The patient tolerated the cesarean delivery well and remained free of neurologic complications postpartum.

## Discussion

These three cases demonstrate the spectrum of pregnancy outcomes in patients with moyamoya disease following prior cerebral revascularization and underscore the importance of individualized, multidisciplinary care. Although all three patients entered pregnancy neurologically stable, their clinical courses diverged significantly based on comorbid conditions and the development of hypertensive complications.

Two patients experienced favorable maternal and neonatal outcomes despite complex medical histories, including pregestational diabetes, multifetal gestation, Chiari malformation, and inherited thrombophilia. In these cases, close surveillance, proactive adjustment of medical therapy, and coordinated management among maternal–fetal medicine, neurology, and anesthesia facilitated stable hemodynamics and timely delivery. These outcomes suggest that prior revascularization surgery and careful longitudinal neurologic follow-up may mitigate—but do not eliminate—pregnancy-associated cerebrovascular risk. 3

In contrast, the fatal outcome in Case 2 underscores the potentially catastrophic interaction between moyamoya pathology and hypertensive disorders of pregnancy. Superimposed preeclampsia with severe features likely exacerbated underlying cerebral vascular fragility, culminating in a large intraparenchymal hemorrhage despite prompt recognition and intervention. 4 This case reinforces that hypertensive crises may represent the most significant modifiable risk factor in pregnant patients with moyamoya disease and that even aggressive inpatient monitoring does not fully eliminate the risk of hemorrhagic stroke.

Delivery planning in moyamoya disease remains controversial. Historically, cesarean delivery or operative vaginal delivery has been favored to reduce Valsalva-associated increase in intracranial pressure. 5 However, existing literature suggests no definitive difference in stroke rates between vaginal and cesarean delivery. 6 In our series, delivery mode was individualized based on obstetric indications, fetal presentation, and maternal stability rather than moyamoya disease alone. This approach aligns with emerging evidence supporting tailored decision-making rather than routine surgical delivery.

Antiplatelet and anticoagulation strategies in pregnancy for patients with moyamoya disease remain variable and are often guided by prior neurologic history and specialist preference rather than high-quality evidence. Current recommendations are largely extrapolated from neurologic society guidelines rather than obstetric-specific evidence. 7 In our series, management ranged from low-dose aspirin to prophylactic low-molecular-weight heparin. While anticoagulation may theoretically reduce thrombotic risk in the setting of arterial stenosis or thrombophilia, its role in preventing ischemic events during pregnancy in moyamoya disease remains unclear. 8 Similarly, optimal blood pressure targets have not been formally defined in this population, though avoidance of both severe hypertension and hypotension is likely critical to maintaining adequate cerebral perfusion.

Collectively, these cases emphasize several clinical priorities: rigorous blood pressure control, early evaluation of new neurologic symptoms, multidisciplinary coordination, and individualized delivery planning. Given the rarity of moyamoya disease in pregnancy and the absence of standardized management guidelines, continued reporting of maternal outcomes is essential to inform risk stratification and optimize care for this high-risk population.
